# Multilayered Mesoporous Composite Nanostructures for Highly Sensitive Label-Free Quantification of Cardiac Troponin-I

**DOI:** 10.3390/bios12050337

**Published:** 2022-05-14

**Authors:** Mohsen Saeidi, Mohammad Ali Amidian, Sana Sheybanikashani, Hossein Mahdavi, Homayoon Alimohammadi, Leila Syedmoradi, Fatemeh Mohandes, Ali Zarrabi, Elnaz Tamjid, Kobra Omidfar, Abdolreza Simchi

**Affiliations:** 1 Department of Materials Science and Engineering, Sharif University of Technology, Tehran 1458889694, Iran; saeidimohsen@gmail.com (M.S.); mohammad.amidian@sharif.edu (M.A.A.); sana.sheybani78@sharif.edu (S.S.); hosein.mahdavi@sharif.edu (H.M.); h.alimohammadi@sharif.edu (H.A.); f.mohandes@sharif.edu (F.M.); 2 Biosensor Research Center, Endocrinology and Metabolism Molecular-Cellular Sciences Institute, Tehran University of Medical Sciences, Tehran 1458889694, Iran; l-syedmoradi@razi.tums.ac.ir; 3 Endocrinology and Metabolism Research Center, Endocrinology and Metabolism Research Institute, Tehran University of Medical Sciences, Tehran 1458889694, Iran; 4 Biomedical Engineering Department, Faculty of Engineering and Natural Sciences, Istinye University, İstanbul 34396, Turkey; ali.zarrabi@istinye.edu.tr; 5 Department of Nanobiotechnology, Faculty of Biological Sciences, Tarbiat Modares University, Tehran 1458889694, Iran; tamjid@modares.ac.ir; 6 Institute for Nanoscience and Nanotechnology, Sharif University of Technology, Tehran 1458889694, Iran

**Keywords:** cardiac troponin-I, electrochemical immunosensors, gold and silver decorated ZIF-67, heart attack risk

## Abstract

Cardiac troponin-I (cTnI) is a well-known biomarker for the diagnosis and control of acute myocardial infarction in clinical practice. To improve the accuracy and reliability of cTnI electrochemical immunosensors, we propose a multilayer nanostructure consisting of Fe_3_O_4_-COOH labeled anti-cTnI monoclonal antibody (Fe_3_O_4_-COOH-Ab_1_) and anti-cTnI polyclonal antibody (Ab_2_) conjugated on Au-Ag nanoparticles (NPs) decorated on a metal–organic framework (Au-Ag@ZIF-67-Ab_2_). In this design, Fe_3_O_4_-COOH was used for separation of cTnI in specimens and signal amplification, hierarchical porous ZIF-67 extremely enhanced the specific surface area, and Au-Ag NPs synergically promoted the conductivity and sensitivity. They were additionally employed as an immobilization platform to enhance antibody loading. Electron microscopy images indicated that Ag-Au NPs with an average diameter of 1.9 ± 0.5 nm were uniformly decorated on plate-like ZIF-67 particles (with average size of 690 nm) without any agglomeration. Several electrochemical assays were implemented to precisely evaluate the immunosensor performance. The square wave voltammetry technique exhibited the best performance with a sensitivity of 0.98 mA mL cm^−2^ ng^−1^ and a detection limit of 0.047 pg mL^−1^ in the linear range of 0.04 to 8 ng mL^−1^.

## 1. Introduction

For decades, the high death rate due to cardiovascular diseases (CVDs with 32% of all global deaths [1]) and the burden cost associated with it demonstrate the necessity for developing more efficient diagnostic gadgets. Acute myocardial infarction (AMI) accounts for about 85% of deaths from CVDs [2]. Hence, the risk prediction by diagnosing biomarkers such as creatine kinase MB (CK-MB), cardiac troponins (cTn), myeloperoxidase (MPO), and myoglobin is beneficial. Human troponin-I (cTnI) antibodies, 23 kDa protein composed of 210 amino acids, take the advantage of high selectivity for the AMI diagnosis [3]. Therefore, developing reliable, sensitive, easy-to-use, and cost-effective immunosensors to quantify the cTnI concentration in real samples with a low detection limit has a high demand for the CVDs management [3,4,5,6].

Over the past decades, different analytical methods based on fluorescence [7], colorimetric [8], surface plasmon resonance [9], enzyme-linked immunosorbent assay (ELISA) [10], and electrochemical techniques [11] have been employed. Among the existing techniques for detection of cTnI, electrochemical immunosensors pay the way for the development of point-of-care (POC) devices with an excellent sensitivity and selectivity, low operating costs, and reliable response [12].

We have recently proposed a sandwich-type electrochemical immunosensor for determining cTnI. Two-dimensional (2D) Zn-MOF/Fe_3_O_4_-COOH/Thi nanocomposites as the electrochemical transducer and an anti-cTnI polyclonal antibody as a bioreceptor were developed, demonstrating a sensitivity of 5.26 µA mL ng^−1^ cm^−2^, detection limit (LOD) of 0.9 pg mL^−1^, wide linear range, and high specificity [3]. Zhao et al. [6] designed an electrochemical immunosensor with a signal transducer of Au nanoparticle (NP)-decorated 2D MOF (Co-BDC)/molybdenum disulfide (MoS_2_) hybrids. Herein, dendritic PtCu alloyed NPs on MoS_2_ functioned as the immobilizers for anti-cTnI antibodies to catalyze the reduction in hydrogen peroxide (H_2_O_2_) with reasonable stability, sensitivity (12.16 µA mL ng^−1^), and low LOD (3.02 fg mL^−1^). Cen et al. [4] suggested that fluffy-like AuPtPd trimetallic-alloyed nanodendrites (AuPtPd FNDs) revealed a wide linear range (0.01–100.0 ng mL^−1^) towards cTnI, due to their excellent specific surface area, immobilizing ability, and electrocatalytic activity. In addition, Zhao et al. [13] employed polypyrrole (PPY) to amplify the signal and strengthen the mechanical stability in combination with PdCuPt NPs and dice-shaped cobalt square cages (DCSCs) for the cTnI quantification. They claimed hierarchical porous DCSCs augmented the mass transfer as well as PdCuPt NPs’ adhesion and distribution on the surface. Their results showed an extremely wide dynamic range (0.050 pg mL^−1^ to 1 μg mL^−1^) and low LOD (0.016 pg mL^−1^). Such studies prove that porous materials can enhance the immunosensor figures of merit, thus employing metal–organic frameworks (MOFs) associated with noble metals in electrochemical sensors is highly demanded.

MOFs are a promising class of synthetic materials comprised of two main components, metal ions or clusters and an organic ligand assembling the structure together [14]. These porous materials come with a variety of inherent advantages such as rich metal active sites, high porosity, diverse structures, and tunable properties [15]. Due to these unique features, MOFs have been widely used in different areas such as catalysis, drug delivery, separation, gas storage, sensors, etc. [16,17,18]. However, the application of single-phase MOFs in electrochemical sensors has been limited due to the poor inherent conductivity of these materials [15]. To overcome this shortcoming, MOF-based composites with higher conductivity have been developed. MOFs have been incorporated with various conductive and electroactive materials including carbon materials such as graphene [19], carbon nanotubes [20], and noble metal NPs (e.g., Ag [21] and Au [22]). Among MOFs, zeolitic imidazolate frameworks (ZIFs) have gained massive recognition in the past few years as a subgroup of MOFs. ZIFs are made of transition metal ions such as Co, Zn, Fe, etc., and deprotonated imidazole as the linker [23]. ZIF-67, as an example, is formed from clusters of cobalt ions (Co^2+^) and 2-methylimidazole [24]. ZIFs show an excellent catalytic activity as the functional groups on the ligand and the incompletely coordinated metal ions act as catalytically active sites. Their large pore volumes, high surface area, and thermal stability make it a suitable support for the diffusion of species [25]. Pan, et al. [26] modified a ZIF-8 by Au NPs and ordered mesoporous carbon (OMC) for the detection of carcinoembryonic antigens. Au@ZIF-8/OMC/GCE exhibited a high surface area (due to ZIF-8) and conductivity (due to OMC and Au NPs), increasing the amount of antibody loaded on the electrode surface and facilitating charge transfer through the interface of the electrode and electrolyte. They asserted that acceptable stability and reproducibility were achieved using such an electrode. Thus, anchoring transition noble metals such as Au and Ag NPs on the porous structure can enhance the electrochemical performance of electrodes.

In this paper, a sandwich-type label-free electrochemical immunosensor was designed for the detection of cTnI using screen-printed carbon electrodes (SPCEs) modified by gold and silver bimetallic NP (Au-Ag NP)-decorated ZIF-67. Au-Ag NPs were successfully electrodeposited on ZIF-67/SPCEs to improve the conductivity of electrodes as well as antibody loading. Fe_3_O_4_-COOH was also employed to isolate the cTnI from specimens and augment the electrode conductivity. Electrochemical properties of immunosensors were boosted by the synergistic effect of Au-Ag NPs and ZIF-67. To the best of our knowledge, the designed immunosensor exhibited the highest sensitivity (0.98 mA mL cm^−2^ ng^−1^) among reported electrochemical immunosensors for cTnI quantification and reached the low LOD in a relatively wide linear range, which makes it suitable for real world applications.

## 2. Materials and Methods

### 2.1. Materials

CoCl_2_·5H_2_O, 2-methylimidazole, polyvinylpyrrolidone (PVP, MW = 40,000 Da), disodium phosphate, potassium chloride, sodium chloride, sodium nitrite, potassium hexacyanoferrate (II and III), Bovine Serum Albumin (BSA), Phosphate buffered saline (PBS), N-hydroxysuccinimide (NHS), 1-ethyl-3-(3-dimethylaminopropyl) carbodiimide (EDC), 11-mercaptoundecanoic acid 95% (MUA), and Cardiac troponin I were purchased from Sigma Aldrich (USA). All the reagents were of analytical grade and used without further purification. Ultrapure deionized water (DI) was supplied by a laboratory water purification system (~18.2 MΩ cm).

### 2.2. Synthesis and Conjugation of Antibody on Fe_3_O_4_ NPs

Superparamagnetic Fe_3_O_4_ nanoparticles were prepared by the chemical co-precipitation method [27]. The nanoparticles were then functionalized with carboxyl groups using citric acid under refluxing conditions in a nitrogen atmosphere [28]. The total mass of the product was 500 mg with a yield of ~7%. To modify the Fe_3_O_4_-COOH nanoparticles with monoclonal anti-cardiac troponin I antibody (Ab_1_), 20 mg NPs were added to 1 mL of the Ab_1_ solution containing EDC/NHS with a ratio of 1:2 ratio. The suspension was then sonicated at room temperature for 20 min and the resulting suspension was washed several times with PBS (0.01 M at pH = 7.40) and dispersed in 1 mL buffer containing 20 mM Na_2_HPO_4_ and 300 mM NaCl. Finally, 1 µg Ab_1_ was introduced to the suspension and incubated under a shaking condition at room temperature for 40 min. The residual surface was blocked using bovine serum albumin (3%).

To functionalize superparamagnetic nanoparticles with cTnI, 15 µL Fe_3_O_4_-COOH-Ab_1_ (80 mg mL^−1^) was introduced to the cTnI solution in PBS (100 µL, pH = 7.40) and incubated for 50 min under shaking. The concentration of cTnI solution was in the range of 0.04 to 10 ng mL^−1^. The nanoparticles were then separated by centrifugation at 4000 rpm for 10 min. Then, they were washed and resuspended in 10 µL of PBS.

### 2.3. Synthesis of Noble Metal Decorated MOFs

First, ZIF-67 was synthesized by the chemical method [29]. Briefly, 520 mg CoCl_2_ was dissolved in 40 mL methanol. Separately, a solution of 600 mg PVP and 2.63 g of 2-methylimidazole in methanol (40 mL) was prepared. The solutions were mixed under stirring and aged for 2 days at room temperature. The precipitate was sequentially washed thoroughly with methanol and deionized water three times and then lyophilized at −50 °C and 29 mTorr for 48 h (CHRIST, Alpha 2-4 LDplus, Osterode am Harz, Germany). Almost 400 mg of the synthesized ZIF-67 was obtained with a yield of ~13%.

Au/Ag modification of ZIF-67 was carried out by two-step electrodeposition after forming a thin layer on a screen-printed carbon electrode (SPCE with the surface area of 0.126 cm^2^, DRP-C110, DropSens, Netherlands) by drop-casting 10 µL of ZIF-67 in methanol with a concentration of 10 mg mL^−1^. To carry out electrodeposition, a mixture of 1 mM HAuCl_4_ and 1 mM AgNO_3_ with 100 mM KNO_3_ as a supporting electrolyte was used. Au-Ag nuclei were formed on ZIF-67/SPCE at a constant potential −1.3 V for 5 s. Afterward, cyclic voltammetry (CV) was employed in a potential window of −1.1 to 0.3 V with a scan rate of 200 mV s^−1^ for 15 cycles (Figure S1 in Supplementary Materials). A similar procedure was used for electrodeposition of Au and Ag NPs on ZIF-67/SPCE.

### 2.4. Fabrication of Troponin-I Immunosensor

To covalently bond the antibody to the surface, carboxylic groups were developed at the surface of the electrode by drop-casting of 10 μL MUA in ethanol (20 mM) followed by incubation at room temperature overnight. Polyclonal anti-cardiac troponin I antibody (Ab_2_) was then introduced to allow amine to carboxylic interactions. The electrode was then washed by PBS to remove unbound chemicals. Finally, 5 µL Fe_3_O_4_-COOH-Ab_1_-cTnI suspension was dropped onto the Ab_2_/Au-Ag@ZIF-67 modified SPCE and incubated for 45 min incubation at room temperature. The immunosensor was rinsed by PBS before electrochemical measurements [30].

### 2.5. Materials Characterization Techniques

***Microscopy.*** Field emission scanning electron microscopy (FE-SEM; Teskan-MIRA3, Czech Republic) at an accelerating voltage of 15 kV equipped with an energy-dispersive X-ray spectrometer (EDS, Oxford) was utilized. High-resolution transmission electron microscopy (HR-TEM, JEOL JEM-ARM200CFEG UHR-TEM) equipped with an EDS spectrometer at an accelerating voltage of 200 kV was carried out.

***Phase analysis.*** Phase analysis was performed by X-ray diffraction (XRD) using a PANalytical diffractometer (Netherlands) with Cu Kα radiation (λ = 1.54 Å) and the angular scan rate of 0.05°/s.

***X-ray photoelectron spectroscopy***. XPS was carried out on a PHI Quantum 2000 instrument (Chanhassen, MN, USA). All spectra were acquired using an Al monochromatized source (15 kV, 10 mA) with a spot size of 500 µm and a resolution of 0.64 eV.

***Specific surface area and pore size.*** The specific surface area (SSA) was determined based on the Brunauer–Emmett–Teller (BET) method by measuring nitrogen adsorption–desorption isotherms (at 77 K). A Micrometric TriStar Plus II analyzer was employed, and the samples were outgassed under 1 mPa at 100 °C for 24 h. The pore size distribution (PSD) was determined by the Barrett–Joyner–Halenda (BJH) method.

***Fourier transform infrared spectroscopy.*** The FT-IR spectrum was acquired by a Perkin Elmer spectrophotometer (USA) using KBr pellets in the range of 400–4000 cm^−1^.

### 2.6. Performance Assessment of Immunosensor

***Voltammetry.*** All electrochemical measurements were performed in a three-electrode configuration using an Autolab potentiostat/galvanostat 302 N (Metrohm, Herisau, Switzerland). The performance of the immunosensor was investigated using four methods including CV, differential pulse voltammetry (DPV), and square wave voltammetry (SWV). Cyclic voltammetry (CV) was carried out in [Fe(CN)_6_]^3-/4-^ solution (5 mM in 0.01 M PBS (pH 7.40) with 0.1 M KCl as a supporting electrolyte). DPV and SWV experiments were conducted at various modulation amplitudes and frequencies, respectively. All experiments were implemented at room temperature (25 °C).

***Electrochemical impedance spectroscopy.*** EIS was performed at zero OCP with a superimposed alternating potential of 5 mV amplitude in a frequency range of 0.1–10^5^ Hz. The experiment was performed in a 5 mM [Fe(CN)_6_]^3-/4-^ solution with 0.01 M PBS (pH 7.40) and 0.1 M KCl.

***Separation of cTnI.*** The isolation of cTnI from the specimen was achieved at room temperature using gentle stirring. Fundamentally, different concentrations of cTnI were prepared. Then, 15 µL of Fe_3_O_4_-COOH-Ab_1_ (80 mg mL^−1^) was introduced to each amount of specimen (100 µL). The resulting product was incubated at room temperature for 50 min with continuous shaking. After completing the attachment process of Fe_3_O_4_-COOH-Ab_1_ to cTnI, the resultant mixture was separated, washed, and resuspended in 10 µL of PBS. Various concentrations of standard cTnI solutions ranging from 0.04 to 10 ng mL^−1^ in PBS were prepared by serial dilutions. Next, 5 µL of Fe_3_O_4_-COOH-Ab_1_-cTnI solution was dropped onto the Ab_2_/Au-Ag@ZIF-67 modified SPCE and the final interaction was achieved with Ab_2_ on the surface of electrode. The electrodes were rinsed with PBS (pH 7.40) and exposed to electrochemical measurements after 45 min incubation at room temperature.

***Stability, repeatability, and reproducibility.*** The stability of the Ab_2_/Au-Ag@ZIF-67/SPCE and Fe_3_O_4_-COOH-Ab_1_ was electrochemically evaluated after 14 weeks of storage at 4 °C and compared to that of freshly provided electrodes. The specificity, repeatability, and reproducibility of the immunosensor were investigated using the measurement of cTnI in several assay runs.

***Real sample analysis.*** Blood samples were collected from patients and healthy volunteers using Ethylenediaminetetraacetic acid (EDTA) tubes. Then, 100 µL of specimens were exposed to 15 µL of Fe_3_O_4_-COOH-Ab_1_ (80 mg mL^−1^). Subsequently, the cTnI protein was measured in the specimens using the process described in the section “Separation of cTnI”.

## 3. Results and Discussion

### 3.1. Morphological Characterization

**Figure 1:**
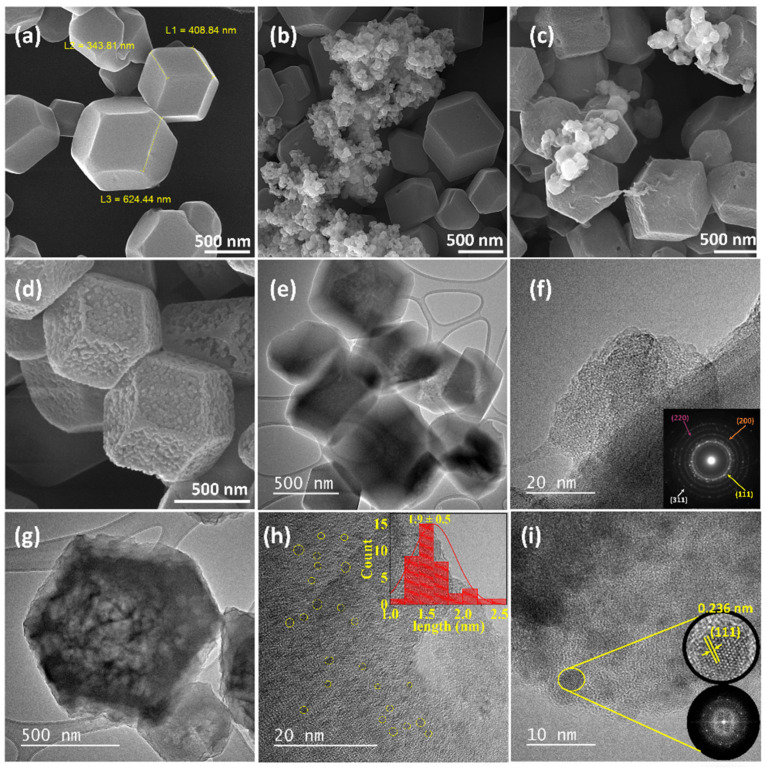
FE-SEM images of (**a**) ZIF-67, (**b**) Au@ZIF-67, (**c**) Ag@ZIF-67, and (**d**) Au-Ag@ZIF-67. HR-TEM of (**e**) and (**f**) ZIF-67 (inset of (**f**) shows the corresponding SAED pattern), and (**g**–**i**) Au-Ag NP@ZIF-67 (insets of (**h**,**i**) show the corresponding nanoparticle size distribution and FFT pattern, respectively).

The morphology of ZIF-67 and size distribution of Au-Ag NPs on ZIF-67 were studied by FE-SEM and HR-TEM as shown in Figure 1a–i and Figure S2a–d. ZIF-67 exhibited a uniform rhombic dodecahedral shape with a narrow size distribution at an average size of ~690 nm (Figure 1a). The smooth surfaces and sharp edges without any agglomeration reveal that an appropriate amount of PVP was used to maintain the morphology of ZIF-67 [31]. The organic linker in the structure of ZIF-67 can efficiently prevent the aggregation of gold and silver nanoparticles, causing several voids in the final product with a rough surface formed by the accumulation of nanoparticles. Figure 1b,c show the morphology of Au@ZIF-67 and Ag@ZIF-67 after electrodeposition of gold and silver nanoparticles on ZIF-67, respectively. Au and Ag nanoparticles were uncontrollably agglomerated in both samples, which is in stark contrast to the uniform electrodeposition of Au-Ag NPs on ZIF-67 (Figure 1d). Au-Ag@ZIF-67 exhibited a more porous and bumpier surface than ZIF-67 with well-distributed nanoparticles on ZIF-67 surfaces. Impressively, the original polyhedral shape of ZIF-67 is completely retained during electrodeposition of Au-Ag NPs together, which can effectively avoid the aggregation of the nanoparticles [32]. These observations indicate that ZIF-67 can be selected as the host for embedding Au and Ag NPs together, which is more favorable for achieving higher electroactive surface area.

The internal structure, morphology and contact form of Au and Ag nanoparticles with ZIF-67 in Au-Ag@ZIF-67 is further investigated by employing HR-TEM. HR-TEM images of ZIF-67 are shown in Figure 1e,f. Obviously, the ZIF-67 crystals were slightly overlapped (Figure 1e). Figure 1g–i demonstrate that each ZIF-67 crystal contained multiple Au-Ag NPs, being fully confined within the ZIF-67 structure. Figure S2a–d display the TEM images of ZIF-67 and Au-Ag@ZIF-67 at different magnifications, further confirming the above discussion. The distance between the NPs and the narrow particle size distribution (with an average size of 1.9 ± 0.5 nm, inset of Figure 1h) suggests that Au and Ag NPs were well isolated from each other during the electrodeposition process (Figure 1h,i). Moreover, the fringes with an interlayer distance of ~0.236 nm for the (111) planes of gold are observed in the HR-TEM image of Au-Ag@ZIF-67 (Figure 1i), which is consistent with the XRD results. The selected area electron diffraction (SAED) pattern of ZIF-67 is shown in the inset of Figure 1f. The diffraction rings reveal a polycrystalline morphology, corresponding well with the (111), (200), (220), and (311) planes of ZIF-67. From the inset of Figure 1i, the fast Fourier transform (FFT) pattern of the selected area showed a sixfold symmetry, which is consistent with the spot pattern of gold nanoparticles [33]. The above results further prove that a close interface does exist between nanoparticles and ZIF-67 crystals, facilitating the electron transfer at the contact interface.

The surface functional groups and defect sites of ZIF-67 can act as anchoring sites for Au and Ag crystals to nucleate and grow on their surface. More importantly, the reason behind the agglomeration of nanoparticles in Au@ZIF-67 and Ag@ZIF-67 can be attributed to the disturbing effect of nanoparticles in the formation of hollow structures on ZIF-67, which is due to the interruption of the outer diffusion of Co [34]. In fact, rapid metal migration in Au@ZIF-67 and Ag@ZIF-67 can induce agglomeration of nanoparticles, thus eventually leading to a bigger size of nanoparticles [35].

The EDS spectra of other samples were depicted in Figure S3a–d. The atomic percent of gold was 1.1 for both Au@ZIF-67 and Au-Ag@ZIF-67 samples while the silver atomic percent was 1.7 and 0.7 for Ag@ZIF-67 and Au-Ag@ZIF-67, respectively. Other elements exhibited almost similar atomic percentages to that of the parent ZIF-67.

**Figure 2:**
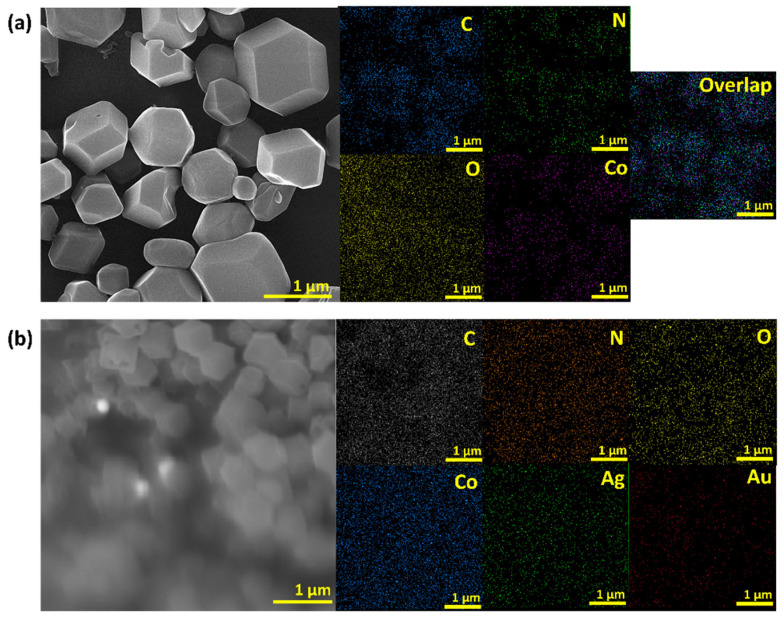
EDS mapping of (**a**) ZIF-67 and (**b**) Au-Ag@ZIF-67.

The EDS elemental mapping analysis of ZIF-67 (Figure 2a) reveals that the uniform distribution of Co particles in the dodecahedral shapes is due to the homogeneous distribution of Co, and C in the whole area. This result suggests that Co does exist in the samples in a highly dispersed state. Figure 2b shows the EDS mapping of Au-Ag@ZIF-67. Au-Ag NPs are uniformly distributed on ZIF-67. The EDS line scanning profile also determines that the amount of Au-Ag NPs in the middle of ZIF-67 are identical but it is much lower than that of Co species (Figure S3e,f).

### 3.2. Chemical and Structural Characterization

**Figure 3:**
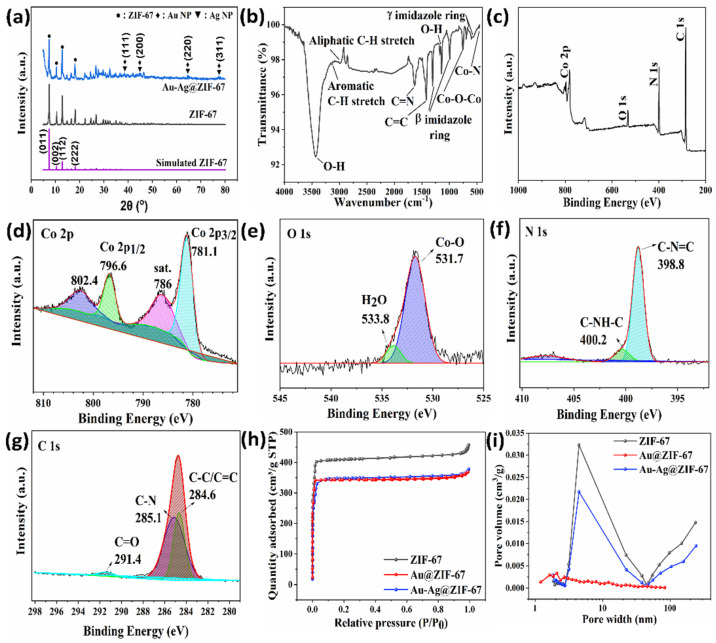
(**a**) XRD patterns of ZIF-67 and Au-Ag@ZIF-67, (**b**) FTIR spectrum of ZIF-67, (**c**–**g**) XPS spectra of ZIF-67, (**h**) N_2_ adsorption/desorption isotherms, and (**i**) Pore size distributions of samples.

X-ray diffraction (XRD) was employed to determine the crystal structure, crystallite size, and phase purity of ZIF-67 and Au-Ag@ZIF-67 (Figure 3a). The peak positions and intensities at 7.4° (011), 10.4° (002), 12.8° (112), and 18.0° (222) are indicative of characteristic diffraction peaks of ZIF-67 (CCDC 67-1073) [36]. The XRD pattern of Au-Ag@ZIF-67 was identical to that of the parent ZIF-67, indicating well the crystallization of ZIF-67 in Au-Ag@ZIF-67. Owning to the low loading and small size of Au and Ag NPs in Au-Ag@ZIF-67, the characteristic diffraction peaks at 38.3° (111), 44.6° (200), 64.6° (220), and 77.4° (311) for Au (JCPDS 89-3697) and Ag nanoparticles are extremely weak (due to the peak broadening and weak signal of the NPs compared to ZIF-67 [37]), being in agreement with FE-SEM and HR-TEM images. As the lattice interspace of the (111) planes for Au and Ag is similar (0.408 and 0.409 nm for Au and Ag, respectively), their XRD patterns are overlaid at 38.3° [38]. The sharp peak at 7.4° in both samples confirms that ZIF-67 with a high crystallinity was formed and its crystal structure remained unchanged during the electrodeposition of Au and Ag NPs [39]. The gold d-spacing for the (111) plane equals 0.235 nm, which is well consistent with HR-TEM results (0.236 nm, Figure 1i). Therefore, with the simple electrodeposition method, the ZIF-67 sample was effectively decorated with Au and Ag NPs. No other diffraction peaks can be observed in the patterns, indicating a high phase purity of the product. The average grain size was further calculated by the Scherrer equation. The average crystallite size of gold and silver nanoparticles was obtained at 17 nm, revealing that Au-Ag@ZIF-67 is attractive as a sensing layer, because the small grain size can enhance sensing properties [40].

Figure 3b displays the FT-IR spectrum of ZIF-67 in the range of 400–4000 cm^−1^. The presence of a vibration band at ∼448 cm^−1^ ascribed to the Co–N stretching bond is indicative of the Co-imidazolate binding [41]. In addition, the Co–O–Co and Co=O stretching modes appeared at 829 and 892 cm^−1^, respectively. The peaks appearing at 695 and 787 cm^−1^ and in the range of 992–1323 cm^−1^ are attributed to out-of-plane ($\gamma_{imidazole\;ring}$) and in-plane bending ($\beta_{imidazole\;ring}$) of the imidazole ring, respectively. Two shoulders at 1561 and 1691 cm^−1^ are ascribed to the stretching and bending N–H vibration of the imidazole ring, respectively [39]. The peaks at 1415, 1625, and 1704 cm^−1^ are attributed to the stretching of C=C, C=N, and N–H bonds in the imidazole group, respectively [42]. The bands at around 2985 and 3123 cm^−1^ are due to the aliphatic and aromatic C-H stretch of the imidazole group, respectively. The absorption peaks at 3428 and 1302 cm^−1^ are attributed to the stretching mode of O-H [39]. The FT-IR spectra combined with SEM, TEM, EDS, and XRD results thus demonstrate ZIF-67 with high purity, and appropriate crystallization was obtained.

To confirm the presence of Co embedded in the ZIF-67 framework, X-ray photoelectron spectroscopy (XPS) was employed. The full survey spectrum (Figure 3c) verifies the presence of Co, C, O, and N elements in the chemical composition of ZIF-67, agreeing with the EDS results. In Figure 3d, two main peaks are observed in the deconvoluted high-resolution spectrum of Co 2p at 781.1 and 796.6 eV, corresponding to Co 2p_3/2_ and Co 2p_1/2_, respectively. In addition, two satellite peaks can be seen at 786 and 802.4 eV, which could be attributed to satellite peaks of Co 2p_3/2_ and Co 2p_1/2_, respectively, indicating that the incorporated Co is in the divalent state (Co^2+^) and also fourfold coordinated by the N from the 2-methylimidazole ligand [43,44]. As for the O 1s spectrum (Figure 3e), two characteristic peaks are observed at 531.7 and 533.8 eV, being ascribed to Co–O and H_2_O, respectively [43,45]. As shown in Figure 3f, the N 1s spectrum reveals two peaks at 398.8 eV and 400.2 eV, which are attributed to pyridinic-N (C=N–C) and pyrrolic-N (C=NH–C), respectively. These observations prove the presence of imidazole ring in ZIF-67 [46]. For the C 1s spectrum (Figure 3g), two peaks are observable at 284.6 and 285.6 eV, which are attributable to C-N and C-C/C=C bonds, respectively [45].

To determine the effect of gold and silver electrodeposition on BET surface area (S_BET_) and pore size distribution (PSD) of ZIF-67, N_2_ adsorption/desorption isotherms were measured as depicted in Figure 3h,i. According to IUPAC classification of adsorption isotherms, all samples exhibited a type I isotherm with abruptly increased adsorption at low relative pressure, which is indicative of micropores [45]. As shown in Figure 3i, although the PSD of Au-Ag@ZIF-67 is comparable to that of the parent ZIF-67, the PSD of Au@ZIF-67 was shifted to the lower pore sizes.

The SSA, pore size, and pore volume of the samples were presented and compared in Table S1. The S_BET_ of ZIF-67 (1705.1 m^2^ g^−1^) decreased after gold electrodeposition (Au@ZIF-67, 1225.3 m^2^ g^−1^). Similarly, the S_BET_ of Au-Ag@ZIF-67 decreased to 1072.5 m^2^ g^−1^, presumably due to the pore blockage at the interface of Au-Ag NPs and ZIF-67 in Au-Ag@ZIF-67, a phenomenon that has been previously reported [38]. This result agrees with the SEM result (Figure 1d). For Au@ZIF-67, gold nanoparticles were aggregated on the ZIF-67 surface (Figure 1b), which resulted in a higher BET surface area compared to that of Au-Ag@ZIF-67. Such augmentation may be due to the agglomeration of Au NPs over the ZIF-67 framework, without intercepting the pores of ZIF-67 [41]. It can be claimed that the smaller particle size of Au-Ag NPs in Au-Ag@ZIF-67 makes them more accessible to the cavities of ZIF-67, resulting in the lower S_BET_ for Au-Ag@ZIF-67 [38]. Hence, the above results are evidence that the highly accessible structure of the ZIF-67 is preserved after electrodeposition of Au-Ag NPs on ZIF-67, and Au-Ag NPs anchored on the pore surface are available for catalytic reactions, facilitating the electron transfer by increasing the interface between the embedded nanoparticles and electrolyte [39,47]. The total pore volume and mean pore width of ZIF-67 (0.67 cm^3^ g^−1^ and 7.2 nm, respectively) are declined by electrodeposition of Au and Au-Ag on ZIF-67 (0.56 cm^3^ g^−1^ and 5.7 nm, respectively, for Au-Ag@ZIF-67). Jiang et al. reported doping Co ions in ZIF-8 decreased the pore diameter from 8 to 5 nm [48], which is consistent with our hypothesis that Au-Ag NPs in Au-Ag@ZIF-67 were located inside the pores. Moreover, for all samples the portion of micropore surface area (S_micro_) and micropore volume (V_micro_) from the total surface area (S_BET_) and pore volume (V_total_), respectively, was dominant (Table S1).

### 3.3. Characterization of Synthesized Fe_3_O_4_-COOH

The size and morphology of the Fe_3_O_4_-COOH nanostructure were examined using TEM. Figure S4a,b demonstrate the TEM image and size distribution of the magnetite nanostructures. In Figure S4a, Fe_3_O_4_ particles were displayed with an average size of 10 nm with spheroid-like structures and narrow size distribution. In Figure S4b, Fe_3_O_4_-COOH was shown with an average size of 12 nm and similar morphology. FTIR spectra of Fe_3_O_4_-COOH nanoparticles and citric acid were shown in Figure S4c, curves a and b, respectively. The characteristic peak of citric acid attributed to the C = O vibration (1705 cm^−1^) shifts to an intense band around 1613 cm^−1^ for Fe_3_O_4_-COOH. The peak displayed at 574 cm^−1^ ascribed to the Fe-O stretching vibration band of Fe_3_O_4_ [28].

### 3.4. Construction of Electrochemical cTnI Immunosensors

**Figure 4:**
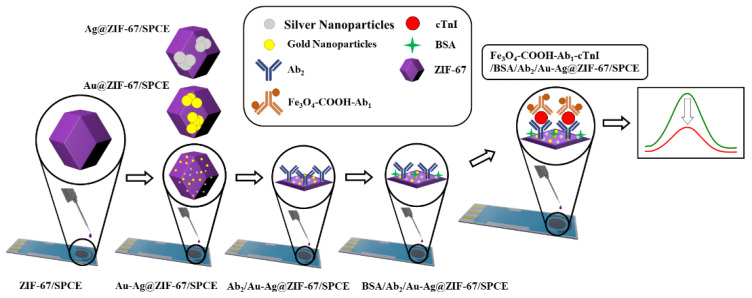
Schematic illustration of the procedure used for preparing the cTnI immunosensors.

In this study, Fe_3_O_4_-COOH-Ab_1_- and Ab_2_/Au-Ag@ZIF-67-modified SPCE were employed to construct a highly sensitive label-free immunosensor, which can be utilized for the quantification of cTnI. Figure 4 demonstrates the process of device assembling and biosensing. It was divided into three main steps. In the first step, the Fe_3_O_4_-COOH nanoparticles were prepared as the two-layer composite. Then, the synthesized nanostructure was conjugated to Ab_1_. The utilization of Fe_3_O_4_-COOH, as the separation tool to label Ab_1_, improved the sensitivity of the assay due to its conducting nature. Alternatively, during the second step, the SPCEs were introduced to multiple modifications including drop-casting of ZIF-67 and electrodeposition of Au-Ag NPs, respectively. It is believed the presence of Au on the electrode surface presented an ideal surface for the efficient covalent immobilization of Ab_2_ and following electrochemical biosensing. Finally, in the third step, Fe_3_O_4_-COOH-Ab_1_ was first subjected to the target analyte, and the complex of nanostructure with analyte was made. This nano-bio complex was dropped on the modified SPCE surface and analyses were then performed when cTnI interacted with Ab_2_ on the sensing surface. The assay procedure was accomplished using different kinds of electrochemical measurements in the presence of potassium ferri/ferrocyanide. According to the design of the immunosensor, the current intensity of electrochemical measurements remarkably decreases with the increase in the cTnI amount of the specimens.

### 3.5. Electrochemical Characterization of Modified SPCE

**Figure 5:**
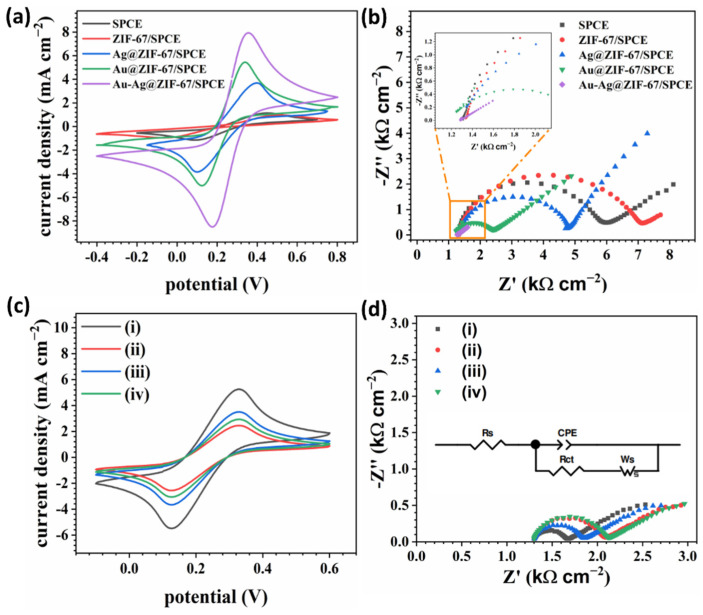
(**a**,**c**) CV curves; (**b**,**d**) EIS characterization of the stepwise modified SPCE. All experiments were implemented in a solution of 5 mM [Fe(CN)_6_]^3/4-^ in 0.01 M PBS containing 0.1 M KCl. Scan rate was 50 mV s^−1^. ((i): Ab_2_/Au-Ag@ZIF-67/SPCE, (ii): BSA/Ab_2_/Au-Ag@ZIF-67/SPCE, (iii): Fe_3_O_4_-COOH-Ab_1_ added to BSA/Ab_2_/Au-Ag@ZIF-67/SPCE, (iv): Fe_3_O_4_-COOH-Ab_1_-cTnI/BSA/Ab_2_/Au-Ag@ZIF-67/SPCE).

Cyclic voltammetry (CV) and electrochemical impedance spectroscopy (EIS) methods were employed to characterize the successive fabrication procedure of the cTnI immunosensors. Figure 5a,c illustrate the CV curves of the stepwise preparation of the sensor in a solution containing 0.1 M KCl and 5 mM Fe(CN)_6_^3-/4-^ (the amount of redox solution used in all the electrochemical measurements was fixed at 70 μL). After assembling ZIF-67 on the SPCE, the redox peak current (red curve) decreased compared to the bare SPCE (black curve), due to the poor conductivity of the ZIF-67 layer. Notably, due to the higher surface area of ZIF-67, more cavities are accessible for the electrodeposition of noble metals, such as Au and Ag. Therefore, by electrodeposition of Ag (blue curve), Au (green curve), and Au-Ag NPs (violet curve) on the ZIF-67/SPCE, the conductivity and electroactive site density at the interface of the modified electrodes and electrolyte enhanced, resulting in the higher redox peak currents in CV curves (Figure 5a). In addition, the peak potentials were shifted to lower potentials by the Au and Ag electrodeposition on ZIF-67/SPCE and the least peak potential separation came from Au-Ag@ZIF-67/SPCE with 150 mV, indicating a reversible process. Hence, the Au-Ag@ZIF-67/SPCE was selected for the subsequent modifications due to the highest current density and least peak potential separation. As shown in Figure 5c, due to the poor conductivity of Ab_2_ (orange curve) the current density slightly decreased, proving that the antibody was attached to carboxylic groups on the modified electrode. Similarly, with the introduction of the BSA layer (cyan curve) the redox peak further declined (compared to Ab_2_/Au-Ag@ZIF-67/SPCE) because of the severe inhibition of charge transfer by the BSA layer. However, by coating the Fe_3_O_4_-COOH-Ab_1_ layer, the current density upsurged since the Fe_3_O_4_-COOH exhibited high conductivity and augmented the electrochemical signal. In fact, this is due to the functional group of citric acids (-COOH), which provides significantly easier accessibility of electrolytes to active the electrode surface [28]. After introducing 0.5 ng mL^−1^ cTnI to the modified immunosensor, the redox peaks extremely decreased, which indicates the successful attachment of cTnI, thus hindering the electron transfer to the electrode surface.

EIS measurements were also accomplished to evaluate the modification steps of the sensor surface (Figure 5b,d). The EIS data agree with the CV results (Figure 5a,c). The EIS is usually composed of two main parts, the semicircle portion at the high-frequency region and the linear portion at the low-frequency portion, indicating the charge transfer and diffusion process, respectively. The real part of impedance (Z′) or the semicircle diameter is primarily used to investigate the performance of the cTnI immunosensors since the Z′ provides information on the applied redox resistance (R_s_) and the charge transfers resistance (R_ct_). The equivalent circuit used for fitting data was shown in the inset of Figure 5d. Since an identical redox probe was used in all experiments, R_s_ was in the range of 160–180 Ω. As depicted in the Nyquist plot (Figure 5b and Table S2), R_ct_ increased from 533.1 Ω for SPCE to 672.2 Ω for ZIF-67/SPCE. However, by electrodeposition of metallic nanoparticles, the resistance significantly dropped to 435.5, 133.4, and almost zero, for Ag, Au, and Au-Ag, respectively. Notably, the R_ct_ of Au-Ag@ZIF-67/SPCE exhibited almost zero, indicating a superior conductive surface with a tiny Warburg line. This result is consistent with CV, SEM, HR-TEM, and BET results. The Nyquist plot of Ab_2_/Au-Ag@ZIF-67/SPCE and BSA/Ab_2_/Au-Ag@ZIF-67/SPCE was displayed in Figure 5c. As it is expected, the semicircle diameter (R_ct_) gradually increased by coating the Ab_2_ and BSA layers on the modified SPCE, revealing the successful immobilizing of the antibody layer and blocking of non-specific sites. These results together proved that the immunosensor was successfully constructed. The electroactive surface area and optimal conditions for the designed immunosensors were investigated and are discussed in the supporting information.

#### 3.5.1. Evaluation of the cTnI Immunosensor

**Figure 6:**
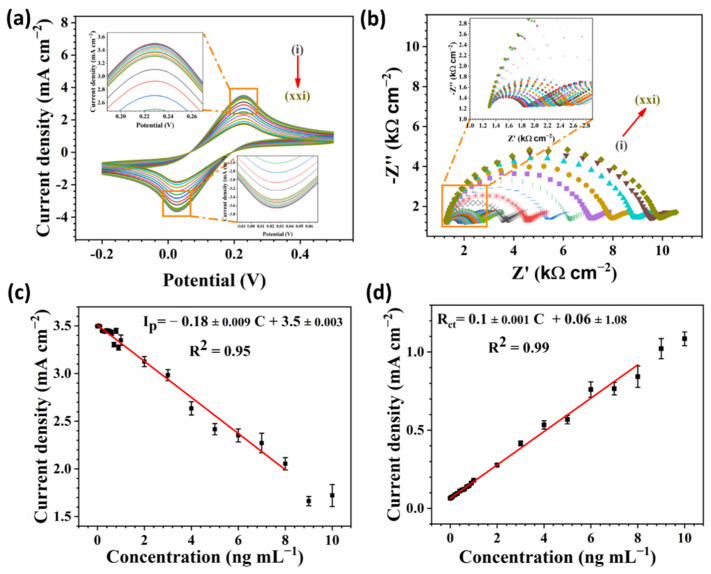
(**a**) CV curves at the scan rate of 50 mV s^−1^; (**b**) EIS curves for various cTnI concentrations from 0 to 10 ng mL^−1^. (**c**,**d**) the corresponding calibration curves with the linear fitting equations for (**a**,**b**), respectively. All experiments were implemented in a solution of 5 mM [Fe(CN)_6_]^3-/4-^ in 0.01 M PBS containing 0.1 M KCl. cTnI concentrations increased in the following order (i): 0, (ii): 0.04, (iii): 0.1, (iv): 0.2, (v): 0.3, (vi): 0.4, (vii): 0.5, (viii): 0.6, (ix): 0.7, (x): 0.8, (xi): 0.9, (xii): 1, (xiii): 2, (xiv): 3, (xv): 4, (xvi): 5, (xvii): 6, (xviii): 7, (xix): 8, (xx): 9, and (xxi): 10 ng mL^−1^.

**Figure 7:**
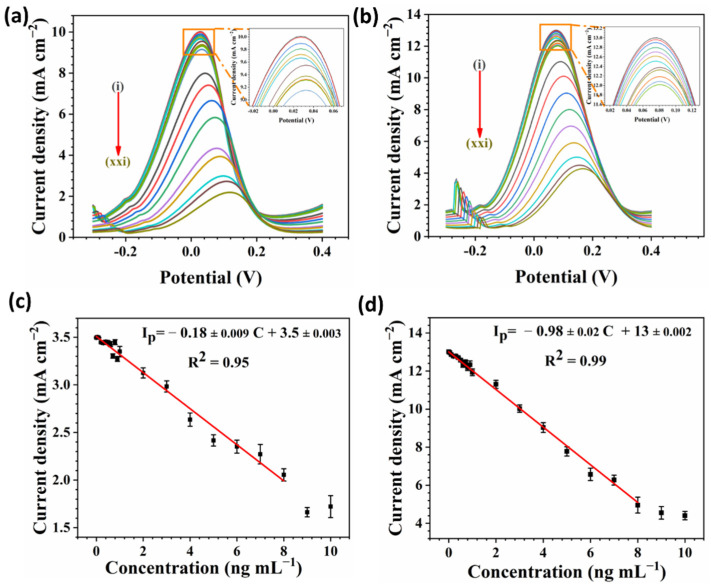
(**a**) DPV curves at the modulation amplitude of 0.25 V; (**b**) SWV curves at the frequency of 15 Hz. (**c**,**d**) the corresponding calibration curves with the linear fitting equations for (**a**,**b**), respectively. All experiments were implemented in a solution of 5 mM [Fe(CN)_6_]^3-/4-^ in 0.01 M PBS containing 0.1 M KCl. cTnI concentrations increased in the following order (i): 0, (ii): 0.04, (iii): 0.1, (iv): 0.2, (v): 0.3, (vi): 0.4, (vii): 0.5, (viii): 0.6, (ix): 0.7, (x): 0.8, (xi): 0.9, (xii): 1, (xiii): 2, (xiv): 3, (xv): 4, (xvi): 5, (xvii): 6, (xviii): 7, (xix): 8, (xx): 9, and (xxi): 10 ng mL^−1^.

To evaluate the performance of the designed immunosensors by changing the cTnI concentration, four different electrochemical techniques were employed: CV, EIS (Figure 6), pulse voltammetry including DPV, and SWV (Figure 7). The cTnI concentrations varied in a range of 0.04–10 ng mL^−1^. Figure 6a depicts the CV curves at various concentrations and clearly, the redox peak current declined by increasing the cTnI concentration. This observation indicates that Fe_3_O_4_-COOH-Ab_1_-cTnI was attached to Ab_2_ and blocked the charge transfer at the electrode/electrolyte interface. However, as illustrated in Figure 6b, the R_ct_ increased with the cTnI concentration, which is consistent with CV results. DPV and SWV curves demonstrated a redox peak decrease with the rising of the cTnI concentration (Figure 7a,b). The redox peak potentials were slightly shifted to higher values due to the blocking effect of Fe_3_O_4_-COOH-Ab_1_-cTnI.

The calibration curves for CV and EIS are shown in Figure 6c,d, respectively. The calibration curves for DPV and SWV techniques are depicted in Figure 7c,d, respectively. The slope, intercept, and coefficient of determination (R^2^) were also presented in the corresponding calibration diagrams. The linear range for all the techniques was from 0.04 to 8 ng mL^−1^. The SWV technique exhibited the highest slope with −0.98 ± 0.02 (R^2^ = 0.99) mA mL cm^−2^ ng^−1^ compared to −0.18 ± 0.009 (R^2^ = 0.95), and −0.8 ± 0.02 (R^2^ = 0.95) mA mL cm^−2^ ng^−1^ for the CV and DPV techniques, respectively. Furthermore, for the EIS technique the R_ct_ slope versus the cTnI concentration was 0.1 ± 0.001 (R^2^ = 0.99) kΩ mL cm^−2^ ng^−1^. The limit of detection (LOD) for each technique was further calculated (S/N = 3), presented, and compared to other reports in Table S4. The LOD for the SWV technique was the minimum with 0.0473 pg mL^−1^ in comparison to 0.0544, 0.0784, and 0.0736 pg mL^−1^ for the DPV, CV, and EIS techniques, respectively. Hence, the SWV technique demonstrated the best performance for the detection of cTnI in this study.

#### 3.5.2. Specificity and Stability of the cTnI Immunosensor

To evaluate the specificity of the designed immunosensor, different proteins including cTnI (500 pg mL^−1^), human serum albumin (HSA, 50 ng mL^−1^), hemoglobin (Hb, 50 ng mL^−1^), C-reactive protein (CRP, 50 ng mL^−1^), cTnC (50 ng mL^−1^) and cTnT (50 ng mL^−1^) were investigated (*N* = 3). As shown in Figure S7a, minimal interference was detected in SWV by replacing cTnI with HSA, Hb, CRP, cTnC, and cTnT. In fact, based on the same principle, a much better electrochemical signal was detected for cTnI compared with other interfering proteins. This result shows a suitable selectivity for the measurement of cTnI.

The stability of Ab_2_/Au-Ag@ZIF-67 modified SPCE and Fe_3_O_4_-COOH-Ab_1_ were also assessed. Different modified SPCEs and the appropriate amount of Fe_3_O_4_-COOH-Ab_1_ were prepared according to what was explained in the experimental section and kept at 4 °C for 14 weeks. As compared with the freshly prepared electrodes, the modified SPCEs exhibited no significant alterations in their current density. The values of relative standard deviation (RSD = 4.3%) and recovery (88.6%) reveal that the presented assay has suitable stability (Figure S7b). The repeatability and reproducibility of the designed immunosensor were also investigated (see Figure S7b,d). The RSD of 1.3% (*N* = 5) and 2.1% (*N* = 6) was obtained for repeatability and reproducibility, respectively. As compared with other studies [3,6,49], the designed immunosensor exhibited a better performance. The results prove that the satisfactory performance of the immunosensor.

#### 3.5.3. Reliability of the cTnI Immunosensor

The response characteristics of the cTnI immunosensor were evaluated by achieving the assay with eight whole blood specimens and comparing the response with those performed using an enzyme-linked immunosorbent assay (ELISA, Monobind Inc, Lake Forest, CA, USA). Table S5 demonstrates the RSD of the assay, which was calculated from three replicated analyses for patient and control samples. An achieved RSD in the range of 0.5 to 1.7% indicates that the present immunosensor is capable of identifying cTnI in real biological specimens with high accuracy, and it is promising for clinical examination.

The concentration of cTnI is directly related to the risk of heart attack in each case, where high concentrations lead to dangerous situations [50]. Hence, determining the concentration of cTnI in a blood sample is of great importance.

## 4. Conclusions

Determining the cTnI concentration in real samples leads to lower mortality rates. A sensitive sandwich-type electrochemical immunosensor via modifying SPCE by Au-Ag NPs decorated on ZIF-67 as an electrochemical platform for immobilization of the cTnI antibody was developed for cTnI determination in real samples. All in all, the designed immunosensor exhibited an extraordinary sensitivity, low detection limit (0.0473 pg mL^−1^ for the SWV technique), and acceptable specificity, reproducibility, and long-term stability. The strategy provides a powerful assay to evaluate antigen–antibody interaction without any sophisticated instrument, thus providing great promise in clinical applications.

## Supplementary Materials

The following supporting information can be downloaded at: https://www.mdpi.com/article/10.3390/bios12050337/s1, Figure S1. CV for electrodeposition of gold and silver nanoparticles on the ZIF-67/SPCE. Figure S2. TEM images of (**a**,**b**) ZIF-67, (**c**,**d**) Au-Ag@ZIF-67. Figure S3. EDS spectra of (**a**) ZIF-67, (**b**) Au@ZIF-67, (**c**) Ag@ZIF-67, and (**d**) Au-Ag@ZIF-67. (**e**,**f**) STEM image and the corresponding EDS line scanning profile of Au-Ag@ZIF-67. Table S1. N2 adsorption/desorption data for the synthesized samples. Figure S4. TEM images of (**a**) Fe_3_O_4_ nanoparticles and (**b**) Fe_3_O_4_-COOH nanostructures. (**c**) FTIR spectra of (**a**) Fe_3_O_4_-COOH (**b**) citric acid. Table S2. Rs and Rct calculated from fitting the experimental data by the equivalent circuit. Figure S5. (**a**–**e**) CV curves at various scan rates for the prepared electrodes. (**f**) The corresponding linear fitting of oxidation peak currents versus the square root of scan rates. All experiments were conducted in a solution of 5 mM [Fe(CN)_6_]^3-/4-^ in 0.1 M PBS containing 0.1 M KCl. Table S3. The electroactive surface area calculated from the linear fitting of oxidation peak currents versus the square root of scan rates. Figure S6. (**a**) DVP curves at the modulation amplitude of 0.1, 0.15, 0.2, and 0.25 V, and (**b**) SWV curves at the frequency of 5, 10, and 15 Hz. The sandwich-type immunosensors of Au-Ag@ZIF-67/SPCEs (0.5 ng mL^−1^ cTnI) were used for all experiments. All experiments were conducted in a solution of 5 mM [Fe(CN)_6_]^3-/4-^ in 0.01 M PBS containing 0.1 M KCl. Figure S7. (**a**) Specificity of the designed immunosensor toward various types of target proteins, (**b**) Stability study for 14 weeks at 4 °C, (**c**,**d**) Repeatability and reproducibility of the modified immunosensor, respectively. All data were presented based on the SWV results. Table S4. Comparison of electrochemical biosensors for detection of cTnI. Table S5. Comparison of CTnI concentrations in whole blood samples detected by our immunosensor and ELISA techniques. References [6,51,52,53].
