# Supplementary material for: Multilayered Mesoporous Composite Nanostructures for Highly Sensitive Label-Free Quantification of Cardiac Troponin-I
Source: Biosensors (Basel). 2022 May 14;12(5):337. doi: 10.3390/bios12050337 (PMC9138364; doi:10.3390/bios12050337)
Supplement: Supplementary file 1 [file biosensors-12-00337-s001.zip › biosensors-1719123-supplementary.pdf]

Supporting Information for

Multilayered Mesoporous Composite Nanostructures for Highly Sensitive Label-free Quantification of Cardiac Troponin-I

Mohsen Saeidi[1], Mohammad Ali Amidian[1], Sana Sheybanikashani[1], Hossein Mahdavi[1], Homayoon Alimohammadi[1], Leila Syedmoradi[2,3], Fatemeh Mohandes[1], Ali Zarrabi[4], Elnaz Tamjid[5], Kobra Omidfar[2,3*], Abdolreza Simchi [1,6*]

[*] Kobra Omidfar, omidfar@tums.ac.ir; Abdolreza Simchi, Simchi@sharif.edu

List of content

S1. Electrodeposition of gold and silver nanoparticles

S2. Morphological studies

S3. Structural and chemical studies

S4. Fe_3O_4-COOH characterizations

S5. Electrochemical studies

S5.1 Electroactive Surface Area Calculations

S5.2 Optimization of cTnI Detection Condition

S5.3 Specificity, stability, reliability, repeatability, and reproducibility

S1. Electrodeposition of gold and silver nanoparticles

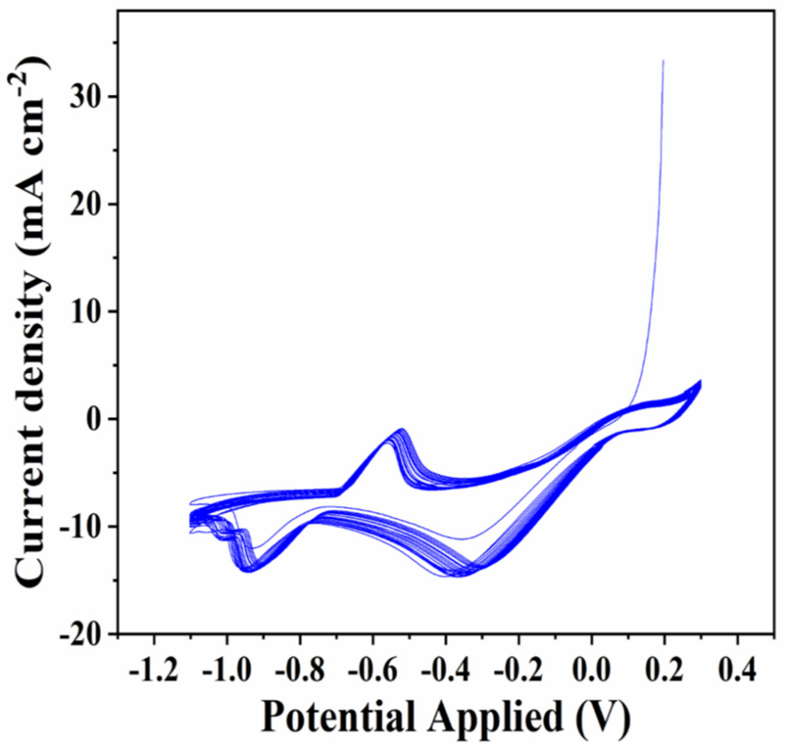

Figure S1. CV for electrodeposition of gold and silver nanoparticles on the ZIF-67/SPCE.

S2. Morphological studies

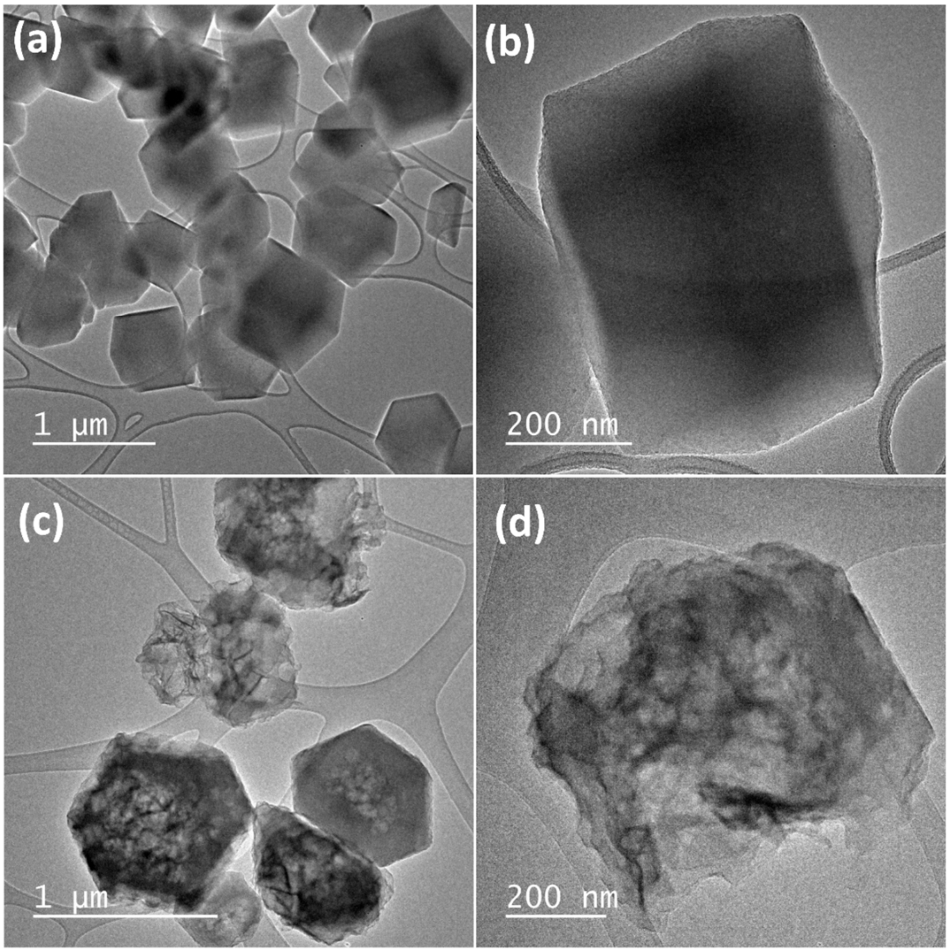

Figure S2. TEM images of **a** and **b** ZIF-67, **c** and **d** Au-Ag@ZIF-67.

S3. Structural and chemical studies

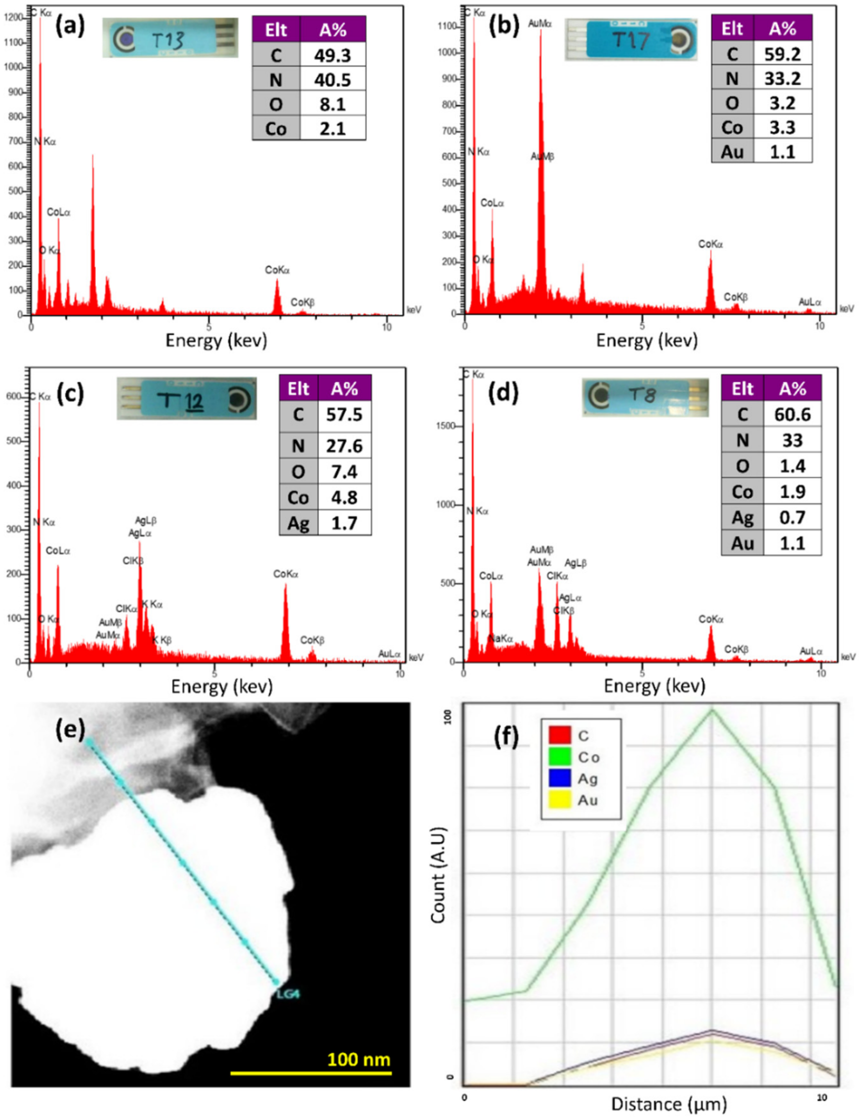

Figure S3. EDS spectra of **a** ZIF-67, **b** Au@ZIF-67, **c** Ag@ZIF-67, and **d** Au-Ag@ZIF-67. **e** and **f** STEM image and the corresponding EDS line scanning profile of Au-Ag@ZIF-67.

Table S1 N_2 adsorption/desorption data for the synthesized samples.

Sample	S_{BET} ($m^2\ g^{-1}$)	S_{ext} ($m^2\ g^{-1}$)	S_{micro} ($m^2\ g^{-1}$)	V_{micro} ($cm^3\ g^{-1}$)	V_{total} ($cm^3\ g^{-1}$)	Mean pore width (nm)
**ZIF-67**	1705.1	17.8	1755.7	0.613	0.667	7.2
**Au@ZIF-67**	1225.3	50.2	1175.1	0.520	0.570	2.1
**Au-Ag@ZIF-67**	1072.5	55.6	1016.9	0.510	0.560	5.7

S4. Fe_3O_4-COOH characterizations

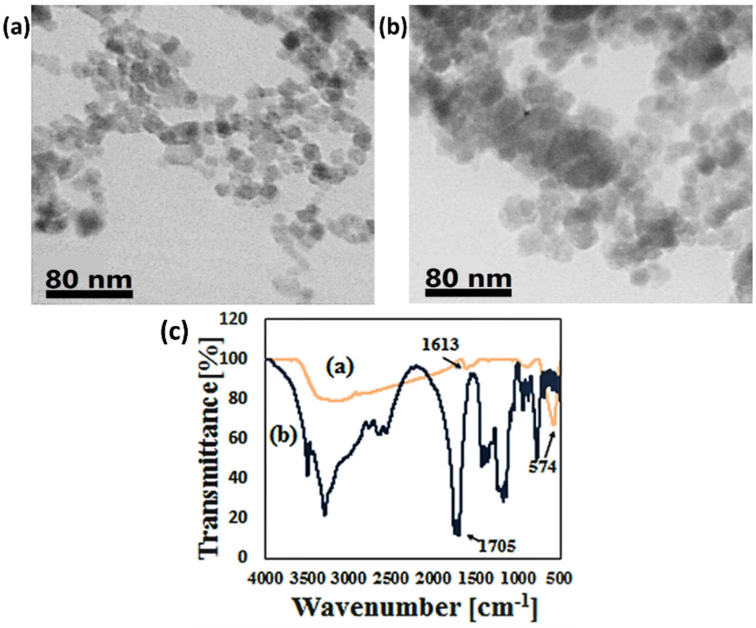

Figure S4. TEM images of (**a**) Fe_3O_4 nanoparticles and (**b**) Fe_3O_4-COOH nanostructures. (**c**) FTIR spectra of (a) Fe_3O_4-COOH (b) citric acid.

S5. Electrochemical studies

Table S2 R_s and R_{ct} calculated from fitting the experimental data by the equivalent circuit.

Substrate	R_s (Ω)	R_{ct} (Ω)
SPCE	164.7	533.1
ZIF-67/SPCE	171	672.2
Ag@ZIF-67/SPCE	168.4	435.5
Au@ZIF-67/SPCE	177.9	133.4
Au-Ag@ZIF-67/SPCE	162.6	≈ 0

S5.1 Electroactive Surface Area Calculations

To further investigate the surface properties of the electrodes, CVs at multiple scan rates were performed. Figure S5a-e show the CV curves of the bare SPCE and all the modified electrodes in a solution of 5 mM $Fe(CN)_6^{3-/4-}$ in 0.01 M PBS containing 0.1 M KCl at several scan rates ranging from 10-300 mV s^{-1}. As shown in Figure S5f, the Randles-Sevcik plot of each electrode is derived from the corresponding CV curves. The anodic peak current (I_{pa}) increased linearly with the square root of the scan rate ($\nu^{1/2}$), suggesting that the redox reaction at the electrode surface was a diffusion-controlled process. The electrochemically active surface area (ECSA) of the electrodes represents both the capacity of the electrode surface to provide conductive path for electron transfer, and the number of catalytically active sites for the electrochemical reactions [1]. For reversible reactions, the value of ECSA can be calculated according to the Randles-Sevcik equation [2]:

$$I_p = (2.69 \times 10^5) n^{3/2} A D^{1/2} \nu^{1/2} C_0$$

where I_p is the peak current (A), n is the number of the electrons participating in the reaction, A is the surface area of the electrode (cm^2), D is the diffusion coefficient of the electroactive species (cm^2 s^{-1}), ν is the scan reate (V s^{-1}), and C_0 is the concentration of the electroactive species in the bulk solution. Based on the slope of the anodic plot of I_p vs. $\nu^{1/2}$, the ECSA was calculated for each electrode (Table S3). The value for SPCE, ZIF-67/SPCE, Au@ZIF-67/SPCE and Ag@ZIF-67/GCE, and Au-Ag@ZIF-67/SPCE is measured as 0.13, 0.07, 0.16, 0.31 and 0.9 cm^2, respectively. The significant increase in the electroactive surface area of Au-Ag@ZIF-67/SPCE (more than seven times higher than the geometrical surface area) can be ascribed to the uniform distribution of nanoparticles on the porous ZIF-67. It can be deduced that the hybrid of ZIF-67 and Au-

Ag NPs, not only could provide facilitated electron transfer pathways, but also a myriad of electroactive sites for the target molecules to be absorbed on the surface.

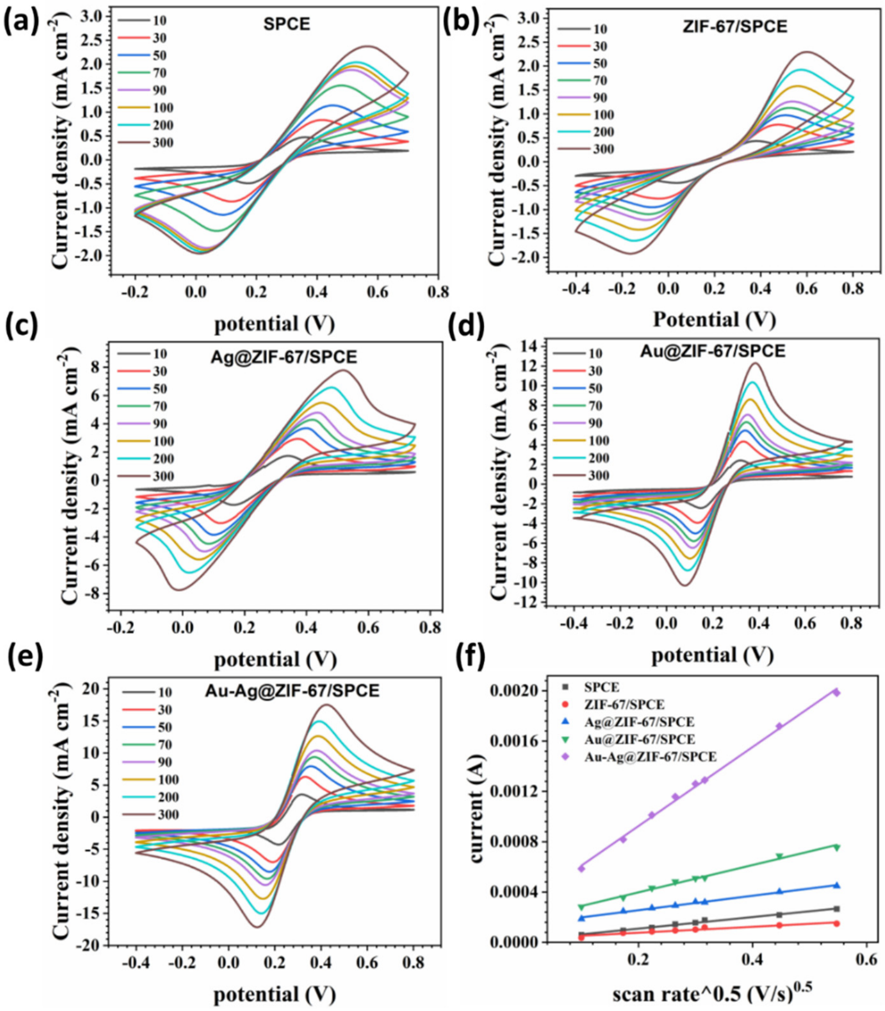

Figure S5. a-e CV curves at various scan rates for the prepared electrodes. **f** the corresponding linear fitting of oxidation peak currents versus the square root of scan rates. All experiments were conducted in a solution of 5 mM $[Fe(CN)_6]^{3-/4-}$ in 0.1 M PBS containing 0.1 M KCl.

Table S3 The electroactive surface area calculated from the linear fitting of oxidation peak currents versus the square root of scan rates

$I_p = m\,\upsilon^{0.5} + a$				
**Substrate**	**m ($A\ s^{0.5}\ V^{-0.5}$)**	**a**	**R^2**	**Electroactive area (cm^2)**
**SPCE**	0.000459	0.000018	0.99	0.13
**ZIF-67/SPCE**	0.000236	0.000029	0.93	0.07
**Ag@ZIF-67/SPCE**	0.000574	0.000141	0.99	0.16
**Au@ZIF-67/SPCE**	0.001081	0.000181	0.99	0.31
**Au-Ag@ZIF-67/SPCE**	0.00314	0.000297	0.997	0.90
Concentration = $5 * 10^{-3}$ (mol L^{-1})			Diffusion coefficient = $6.7 * 10^{-6}$ ($cm^2\ s^{-1}$)	

S5.2 Optimization of cTnI Detection Condition

To determine the maximum sensitivity in this assay, the optimal amount of Fe_3O_4-COOH-Ab_1 and Ab_2 were assessed. First, various amounts of Ab_2 (0.1, 0.2, 0.3, 0.4 and 0.5 μg) were immobilized onto the Au-Ag@ZIF-67 modified SPCE surface. The optimal concentration of the Ab_2 for the assay was detected around 0.3 μg. Following, different volumes of Fe_3O_4-COOH-Ab_1 were analyzed, and 15 μL was ultimately determined as the ideal amount for all electrochemical measurement. The results indicated that the increase in concentration of Fe_3O_4-COOH-Ab_1 leads to decrease in the signal intensity; however, due to the low current intensity change between 15, 18 and 20 μL of Fe_3O_4-COOH-Ab_1, 15 μL was selected as the ideal volume for all electrochemical assays. The appropriate amounts of components were determined based on the SWV results of 500 pg mL^{-1} cTnI. Additionally, the effect of various incubation times (30–60 min) and temperatures (25 and 37 °C) on the rate of interaction between the target with Ab_1 and Ab_2 were also evaluated. The analysis of SWV response exhibited that the best signals found after 40 and 50 min incubation at 25 °C, respectively (data not shown).

To optimize the performance of the immunosensor for detecting cTnI, the various electrochemical parameters were investigated as shown in Figure S6. For optimizing the Au-Ag NPs electrodeposition procedure, the CV experiment was carried out at 5, 10, 15, and 20 cycles (data not shown). The electrode with 15 cycles showed the highest current density and was used for the further experiments. To optimize all parameters the constant concentration of cTnI (0.5 ng ml^{-1}) was used. The current density after 45 min is almost constant, therefore all the experiments were performed after 45 min. Moreover, the modulation amplitude for the DPV experiment was investigated at 0.1, 0.15, 0.2, 0.25 V (Figure S6a) and the optimized modulation amplitude was selected at 0.25 V. The

frequency for the SWV method was further examined at 5, 10, and 15 Hz and at the frequency of 15 Hz, the highest current density was obtained (Figure S6b).

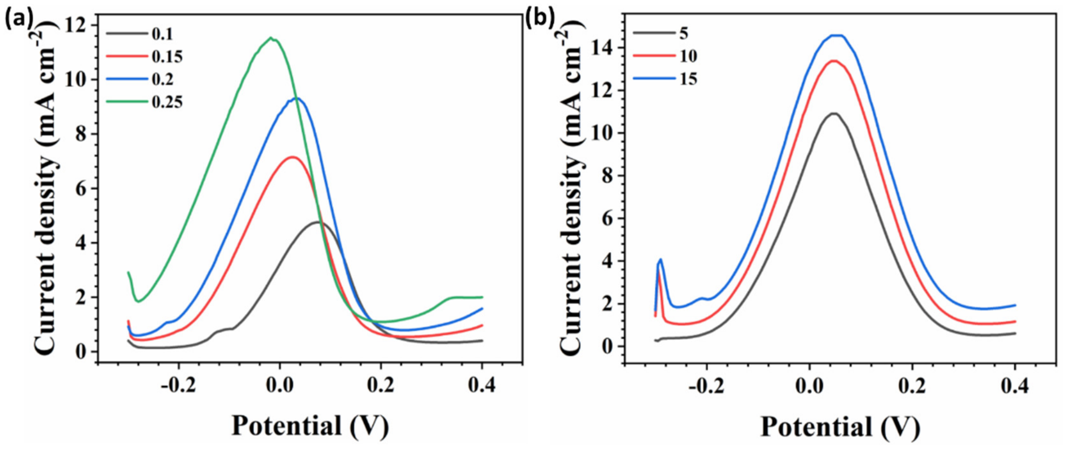

Figure S6. a DVP curves at the modulation amplitude of 0.1, 0.15, 0.2, and 0.25 V, and **b** SWV curves at the frequency of 5, 10, and 15 Hz. The sandwich-type immunosensors of Au-Ag@ZIF-67/SPCEs (0.5 ng mL^{-1} cTnI) were used for all experiments. All experiments were conducted in a solution of 5 mM $[Fe(CN)_6]^{3-/4-}$ in 0.01 M PBS containing 0.1 M KCl.

S5.3 Specificity, stability, reliability, repeatability, and reproducibility

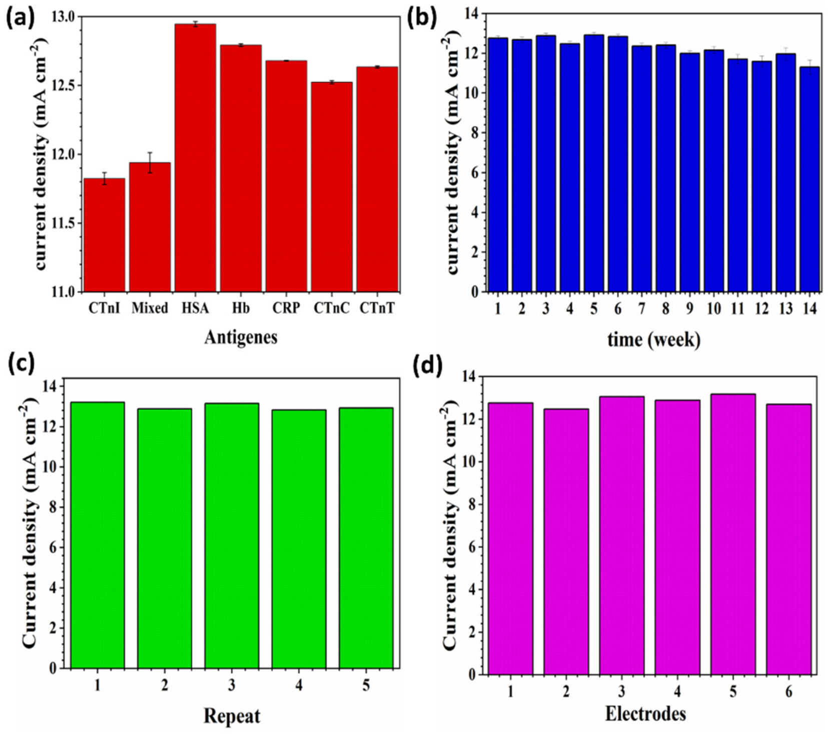

Figure S7. a Specificity of the designed immunosensor toward various types of target proteins, **b** Stability study for 14 weeks at 4 ℃, **c**, and **d** Repeatability and Reproducibility of the modified immunosensor, respectively. All data were presented based on the SWV results.

Table S4 Comparison of electrochemical biosensors for detection of cTnI.

Electrode	Detection Technique	Linear range (pg mL^{-1})	LOD (pg mL^{-1})	Ref.
Au/Co-BDC/MoS2/GCE	CA	0.01-100000	0.00302	[3]
PdCuPt/PPY/DCSC/GCE	CA	0.05-1000000	0. 01603	[4]
Cu3(BTC)2/PANI/SPCE	EIS	1000-400000	800	[5]
aptamer-MoS2 NSs/GCE	EIS	10-1000000	0.95	[6]
CIL-HCNTs/GCE	DPV	10-60000	0.6	[7]
Ag-Au@ZIF-67/SPCE	SWV	40-8000	0.0473	this study
	DPV		0.0544	
	CV		0.0784	
	EIS		0.0736	

Table S5 Comparison of CTnI concentrations in whole blood samples detected by our immunosensor and ELISA techniques.

ELISA results (pg mL^{-1})	Developed biosensor results (pg mL^{-1})	SD	RSD (%)	Recovery (%)
43	42	0.707	1.66	98
52	53	0.71	1.37	98
124	123	0.71	0.57	99
256	258	1.41	0.55	101
396	391	3.54	0.9	99
451	446	3.54	0.79	99
569	560	6.36	1.13	98
687	696	6.36	0.92	101

References

1. Jian, J.M.; Fu, L.; Ji, J.; Lin, L.; Guo, X.; Ren, T.L. Electrochemically Reduced Graphene Oxide/Gold Nanoparticles Composite Modified Screen-Printed Carbon Electrode for Effective Electrocatalytic Analysis of Nitrite in Foods. *Sensors Actuators B. Chem.* **2018**, *262*, 125–136, doi:10.1016/j.snb.2018.01.164.
2. Chen, B.; Zhang, Y.; Lin, L.; Chen, H.; Zhao, M. Au Nanoparticles @metal Organic Framework/Polythionine Loaded with Molecularly Imprinted Polymer Sensor: Preparation, Characterization, and Electrochemical Detection of Tyrosine. *J. Electroanal. Chem.* **2020**, *863*, 114052, doi:10.1016/j.jelechem.2020.114052.
3. Zhao, H.; Du, X.; Dong, H.; Jin, D.; Tang, F.; Liu, Q.; Wang, P.; Chen, L.; Zhao, P.; Li, Y. Electrochemical Immunosensor Based on Au/Co-BDC/MoS2 and DPCN/MoS2 for the Detection of Cardiac Troponin I. *Biosens. Bioelectron.* **2021**, *175*, doi:10.1016/j.bios.2020.112883.
4. Zhao, H.; Cao, L.; Liu, Q.; Tang, F.; Chen, L.; Wang, S.; Li, Y.; Li, Y.; Li, B.; Liu, H. Label-Free Electrochemical Immunosensor Based on PdCuPt/PPY/DCSC as a Signal Amplification Platform for Sensitive Detection of Cardiac Troponin I. *Sensors Actuators B Chem.* **2022**, *351*, 130970, doi:10.1016/j.snb.2021.130970.
5. Gupta, A.; Sharma, S.K.; Pachauri, V.; Ingebrandt, S.; Singh, S.; Sharma, A.L.; Deep, A. Sensitive Impedimetric Detection of Troponin I with Metal-Organic

Framework Composite Electrode. *RSC Adv.* **2021**, *11*, 2167–2174, doi:10.1039/d0ra06665f.

6. Qiao, X.; Li, K.; Xu, J.; Cheng, N.; Sheng, Q.; Cao, W.; Yue, T.; Zheng, J. Novel Electrochemical Sensing Platform for Ultrasensitive Detection of Cardiac Troponin I Based on Aptamer-MoS2 Nanoconjugates. *Biosens. Bioelectron.* **2018**, *113*, 142–147, doi:10.1016/j.bios.2018.05.003.
7. Yan, H.; Tang, X.; Zhu, X.; Zeng, Y.; Lu, X.; Yin, Z.; Lu, Y.; Yang, Y.; Li, L. Sandwich-Type Electrochemical Immunosensor for Highly Sensitive Determination of Cardiac Troponin I Using Carboxyl-Terminated Ionic Liquid and Helical Carbon Nanotube Composite as Platform and Ferrocenecarboxylic Acid as Signal Label. *Sensors Actuators, B Chem.* **2018**, *277*, 234–240, doi:10.1016/j.snb.2018.09.010.
